# A Case Report on Vaginal Melanoma, a Fast, Progressive Disease

**DOI:** 10.7759/cureus.101360

**Published:** 2026-01-12

**Authors:** Mafalda Castro Neves, Daniela Rocha, Rosália Coutada, Agostinho Carvalho, Paula Pinheiro

**Affiliations:** 1 Gynecology and Obstetrics, Unidade Local de Saude Sao Joao, Porto, PRT; 2 Gynecology, Unidade Local de Saúde do Alto Minho, Viana do Castelo, PRT; 3 Gynecologic Oncology, Unidade Local de Saúde do Alto Minho, Viana do Castelo, PRT; 4 Obstetrics, Unidade Local de Saúde do Alto Minho, Viana do Castelo, PRT

**Keywords:** case report, kit mutation, mucosal melanoma, nivolumab, vaginal melanoma

## Abstract

Vaginal melanoma (VM) is a very rare sort of primary vaginal cancer, accounting for very few malignant vaginal diseases, and even less of all types of melanomas. It is a highly aggressive and progressive tumor, with high rates of recurrence and metastasis. We report the case of an 83-year-old woman who presented with abnormal uterine bleeding and was ultimately diagnosed with primary vaginal mucosal melanoma. The case highlights the diagnostic difficulties, the importance of early biopsy and histopathology, including immunohistochemistry, and the challenges in systemic management for mucosal melanoma.

## Introduction

Primary vaginal melanoma (VM) is an extremely rare malignancy, accounting for less than 3% of vaginal cancers and about 1% of all melanomas. Fewer than 500 cases have been reported in the literature [1-4]. It typically affects postmenopausal women and arises most often in the lower third of the vagina, more frequently on the anterior wall in an area of difficult access for inspection. The first real challenge of VM is its asymptomatic nature or the nonspecific symptoms, which can have a major impact on disease progression.

Clinically, VM usually presents as a polypoid, friable, or bleeding mass, and may be pigmented or amelanotic, which often delays clinical suspicion [3,5]. Most clinicians often think of more frequent and benign causes for vulvar/vaginal masses amongst the elderly, such as organ prolapse or cysts [6]. Unlike cutaneous melanoma, the risk factors for VM are poorly defined, and there is no established association with ultraviolet exposure [5]. Vaginal melanomas are more aggressive than their cutaneous counterparts, with 5-year survival rates ranging between 5% and 25% [3-5].

Due to the rarity of this condition, there are limited data regarding treatment and follow-up recommendations. The primary treatment remains surgery with wide local excision of the primary lesion, usually not requiring exenteration, along with sentinel lymph node (SLN) mapping. In locally advanced cases, radiotherapy combined with immunotherapy should be considered. Systemic chemotherapy has shown modest response rates and poor survival outcomes, while newer immunotherapeutic and targeted agents have demonstrated improved results and should be considered in selected cases of VM [4,7-9].

## Case presentation

An 83-year-old woman presented to the Gynecology Department of Unidade Local de Saúde do Alto Minho (ULSAM) in August 2024 with a 2-month history of abnormal uterine bleeding. Her medical history included rheumatoid arthritis (seropositive), hypertension, dyslipidaemia, chronic gastritis, and multiple prior surgeries (hip arthroplasty, appendectomy, cholecystectomy, tonsillectomy). Earlier in 2024, she had undergone excision of a pT1 R0 grade 2 squamous cell carcinoma of the left malar region and a basal cell carcinoma of the lower lip.

**Figure 1:**
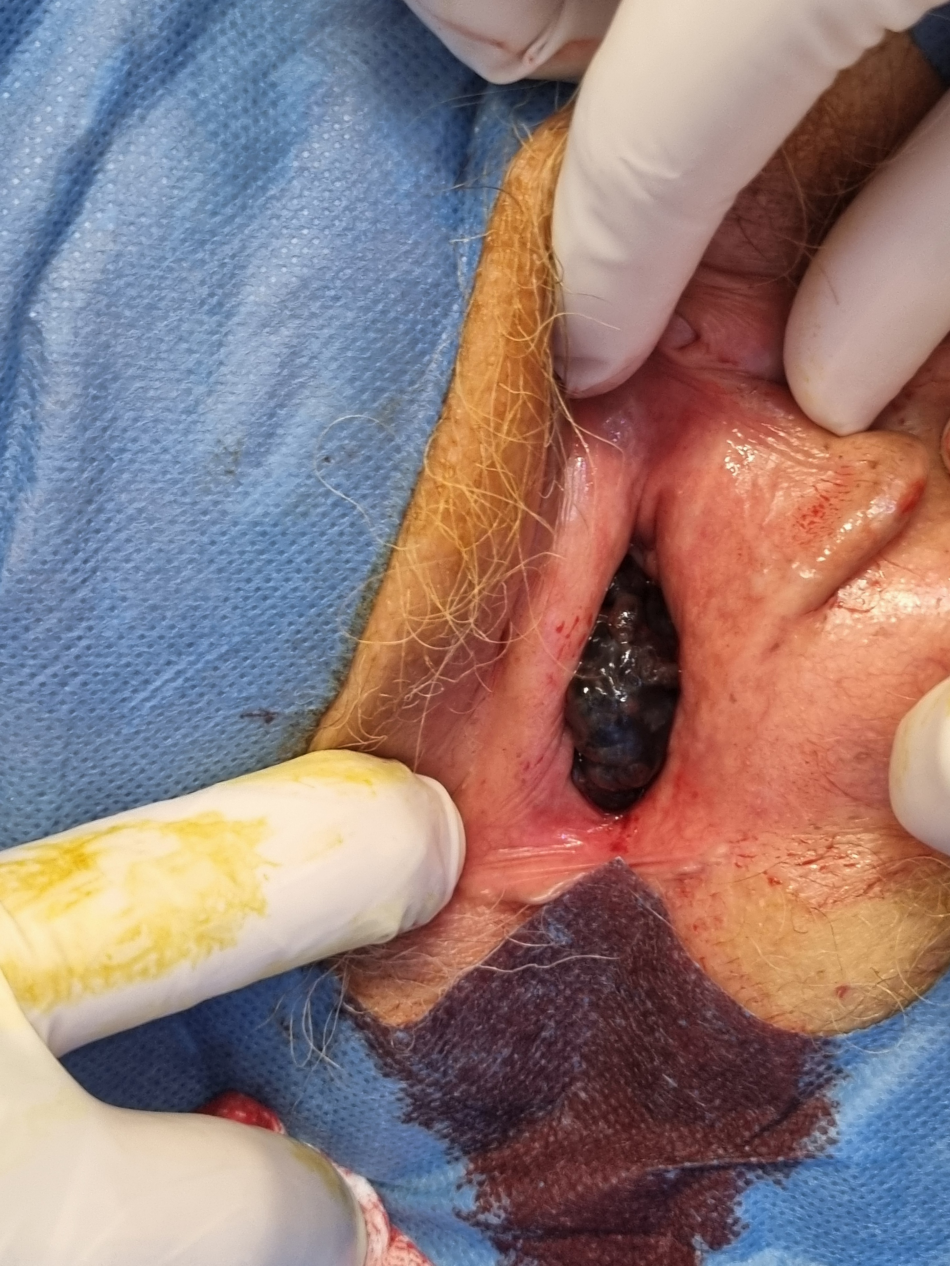
Exophytic, irregular, pigmented vaginal mass visible at the vaginal introitus, involving the lower third of the right lateral vaginal wall at initial clinical presentation

**Figure 2:**
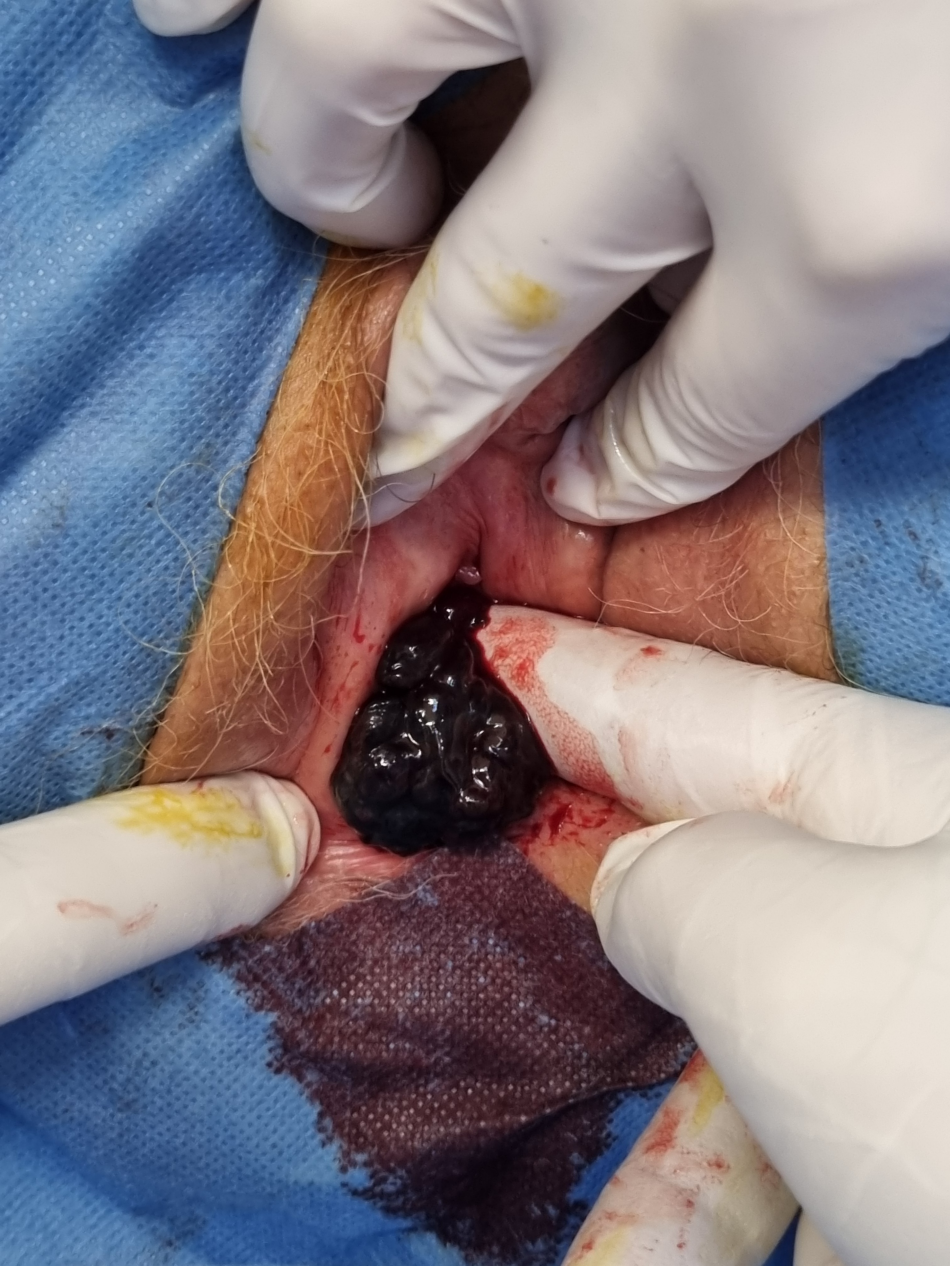
Extension of the vaginal melanoma along the inner vaginal wall, demonstrating an irregular, vegetative lesion with a friable surface

**Figure 3:**
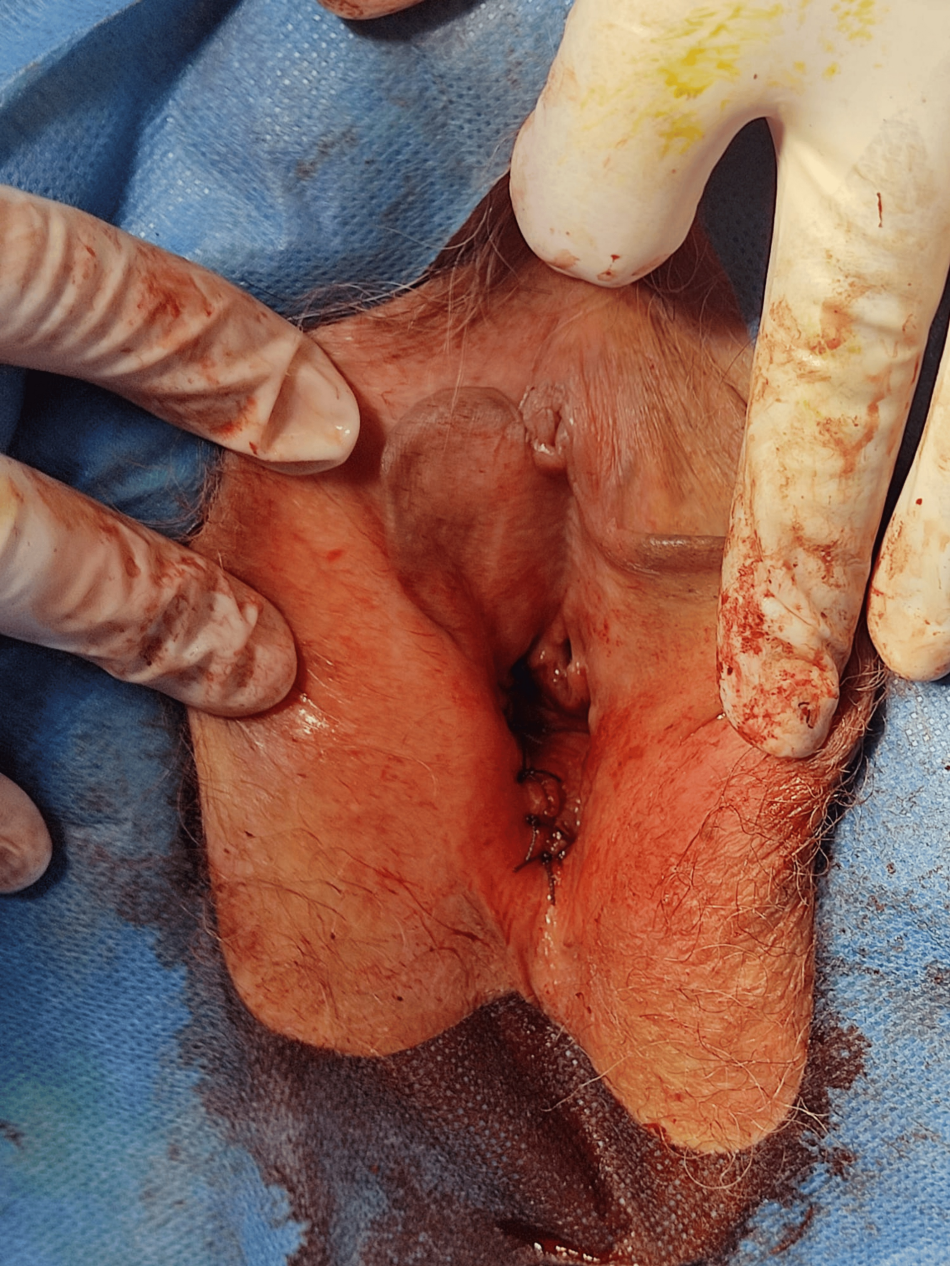
Postoperative appearance of the vagina following surgical excision of the primary lesion and additional satellite lesions

On gynecological examination, a 4-cm cyanotic, irregular, firm, vegetative lesion on the right lateral vaginal wall, in the lower third, was observed (Figures 1-3). No inguinal lymphadenopathy was detected. Because the patient did not tolerate an office biopsy due to pain and bleeding, the team proceeded with surgical excision of the mass; during the surgical procedure, more lesions were found deeper in the vagina on the posterior and left wall near the cervix and were also removed. The specimen was received by the pathology department on 24 September 2024.

Pathology findings

Histopathological examination revealed dermopapillary mucosa extensively infiltrated by malignant melanoma. The tumor consisted of solid sheets of large atypical melanocytic cells displaying rounded nuclei, vesicular chromatin, prominent nucleoli, and scant cytoplasm containing coarse brown pigment. The lesion demonstrated a minimum thickness of at least 15 mm and was ulcerated. There were features suggestive of lymphatic invasion, though no unequivocal vascular invasion was identified. The mitotic index was extremely high, exceeding 150 mitoses per mm², and both the deep and lateral surgical margins were involved by the tumor.

Immunohistochemistry confirmed melanocytic differentiation, with diffuse positivity for S100, HMB45, and Melan-A, and negativity for AE1/AE3. The final diagnosis was malignant melanoma of the vaginal mucosa.

Oncologic workup and molecular studies

The patient was referred to the Portuguese Oncology Institute (IPO Porto) for follow-up and treatment. Initial staging performed in late 2024 showed no distant metastases, but the lesion was deemed unresectable due to its depth and margin involvement. Molecular analysis demonstrated BRAF wild-type status and identified a KIT L576P exon 11 mutation (mutant allele frequency ~68%).

Based on multidisciplinary team (MDT) consensus, systemic immunotherapy with nivolumab (administered every four weeks) was initiated in February 2025. After two cycles, the patient developed worsening vaginal bleeding, pelvic discomfort, and constitutional symptoms. Repeat imaging confirmed local progression. From June 2025 onwards, she experienced progressive systemic decline with fever, weight loss, abdominal and lumbar pain, grade 4 anemia, and renal dysfunction.

Given the presence of a KIT mutation, off-label imatinib was requested and subsequently authorized on 8 May 2025. Treatment was initiated, but clinical improvement was not achieved.

Disease progression and terminal course

By September 2025, the patient demonstrated rapid systemic progression. A PET scan performed on 24 September 2025 showed extensive metastatic disease, with involvement of the vagina (with probable extension to the lower uterus), pelvic lymph nodes, liver, lungs, bone, and mediastinal lymph nodes. Importantly, the scan also revealed new bilateral hypermetabolic cerebral lesions, consistent with intracranial metastases.

She was admitted for IPO (Inpatient Only Procedure) due to symptoms related to newly diagnosed brain metastases, including progressive confusion and headaches. Brain MRI confirmed multiple supra- and infratentorial metastases, associated with surrounding oedema and mass effect; the largest lesion measured approximately 25 mm. Given the extent of metastatic involvement and clinical deterioration, systemic therapy was discontinued. She was started on high-dose corticosteroids (dexamethasone 16 mg/day) for symptomatic relief.

On 2 October 2025, one day after discharge, the patient was brought to the emergency department following six tonic-clonic seizures, requiring midazolam, diazepam, and levetiracetam administered by pre-hospital medical services. On arrival, she was somnolent but no longer convulsing. Neurological decline was attributed to progressive intracranial metastatic disease.

In view of her disseminated metastases, worsening neurological status, and lack of further therapeutic options, she was transitioned to comfort-focused measures. The patient died on 3 October 2025, approximately one year after diagnosis, due to complications associated with cerebral disease metastasis and multiple organ failure.

## Discussion

This case illustrates several features associated with poor prognosis in mucosal (vaginal) melanoma. The patient was elderly, within the commonly described age range for VM (most cases occur in postmenopausal women, typically between 60 and 80 years) but at the older extreme [3]. The tumor presented as a polypoid, bleeding lesion located in the lower third of the vagina - the classical site for VM - and histopathology demonstrated multiple adverse prognostic features: very large thickness (≥15 mm), ulceration, extremely high mitotic index (>150 mitoses/mm²), lymphatic invasion, and positive deep and lateral margins. These histological features portend aggressive local behavior and a higher risk of metastasis [3,5].

Immunohistochemistry was consistent with melanoma lineage (S100, HMB45, and Melan-A positive; AE1/AE3 negative), which supports the diagnosis and is typical for mucosal melanomas [6]. Molecular testing identified KIT L576P (exon 11) and BRAF wild type; KIT alterations are seen more frequently in mucosal than cutaneous melanoma and can be therapeutically actionable [7,8]. While KIT inhibitors, such as imatinib, have shown responses in selected KIT-mutant melanomas, response rates are variable and often short-lived. Responses correlate with the nature of the KIT mutation and other tumor characteristics [7,8]. In this case, imatinib was authorized and used off-label after progression, but clinical benefit was not achieved before further systemic deterioration.

Compared with published series, this case is concordant with several known patterns in VM: older age at presentation, frequent location in the lower vagina, possible amelanotic or pigmented appearance, and poor overall survival. Reported 5-year survival for vaginal mucosal melanoma is generally poor (commonly 5-25%) [3-5]. The presence of symptomatic cerebral metastases late in the course and rapid systemic spread mirrors the aggressive natural history previously described for mucosal melanomas, and the pathologic high mitotic rate and deep invasion likely contributed to the rapid progression despite treatment.

This case highlights several practical points, such as early biopsy and expedited histopathologic evaluation (including IHC and molecular testing), which are critical to guide management and identify potentially targetable mutations. Even when a potentially actionable driver (e.g., KIT mutation) is present, systemic therapy (immunotherapy or targeted therapy) may have limited efficacy in the context of heavy tumor burden, very aggressive histology, and symptomatic CNS disease.

MTD discussion and palliative planning are essential early, particularly in elderly patients with comorbidities and aggressive tumor biology.

## Conclusions

Primary vaginal melanoma remains a rare, aggressive tumour with limited evidence to guide management. The combination of advanced age, deeply invasive tumor, extremely high mitotic index, lymphatic invasion, positive margins, and development of multiorgan metastases, including symptomatic brain lesions, contributed to rapid progression and death despite sequential immunotherapy and targeted therapy. Early recognition, comprehensive pathologic and molecular characterization, and individualized multidisciplinary care remain the cornerstones of management for this challenging disease. Reporting such cases improves collective knowledge about prognosis and therapeutic options in mucosal melanoma.
